# Advances in the Diagnosis of Reproductive Disorders in Female Camelids

**DOI:** 10.3390/ani15192902

**Published:** 2025-10-04

**Authors:** Abdelmalek Sghiri, Michela Ciccarelli, Muhammad S. Waqas, Abelhaq Anouassi, Ahmed Tibary

**Affiliations:** 1 School of Veterinary Medicine, Instituts Agronomique et Vétérinaire Hassan II, Rabat-Instituts, P.O. Box 6202, Rabat 10101, Morocco; 2 College of Veterinary Medicine, Washington State University, Pullman, WA 99164, USAtibary@wsu.edu (A.T.); 3 Advanced Scientific Group, ASG Sweihan, Abu Dhabi P.O. Box 45803, United Arab Emirates

**Keywords:** dromedary, alpacas, llamas, breeding soundness exam, infertility, pregnancy loss

## Abstract

**Simple Summary:**

Camelid reproductive efficiency in traditional rearing systems is low. Reproductive disorders, including those unique to these species, have been identified. This review describes the approach and diagnoses of infertility and subfertility cases in camelids referred to the authors in the last 35 years. A major problem encountered in camels is the lack of a precise reproductive and health history. Ultrasonography, endometrial cytology, bacteriology, and biopsy are the primary techniques used for the examination of the reproductive system. Advanced techniques such as laparoscopy, hysteroscopy, and cytogenetics allow a more precise diagnosis in some cases. These clinical skills are essential for the practitioner.

**Abstract:**

Camelids are increasingly recognized as important livestock species. They are valuable sources of meat, fiber, and milk. Despite their growing popularity, many aspects of their reproductive physiology and pathology remain unclear. Their reproductive performance is reported to be low in many countries. Advances in camelid veterinary care have identified several disorders, some of which are species-specific. This article describes an approach to and the diagnosis of infertility and subfertility cases in alpacas, llamas, and camels referred to the authors over the past 35 years. Ultrasonography, endometrial cytology, and biopsy are the primary diagnostic tools for practitioners. However, laparoscopy, hysteroscopy, and cytogenetics are indicated for cases referred to theriogenologists. The incidence of congenital and acquired reproductive disorders is presented. A high incidence of congenital defects of the reproductive tract is found in South American camelids, which raises concerns about animal welfare. Acquired disorders are similar to those described in other species. Endometritis and endometrosis are major disorders contributing to infertility and early pregnancy loss. However, studies on uterine defense mechanisms and the pathogenesis of these disorders are lacking. Hydrobursitis, a common cause of infertility in dromedary camels, warrants further research. The implications of some contagious diseases (tuberculosis, campylobacteriosis, and brucellosis) in female infertility are discussed. These findings emphasize the importance of including camelid medicine in veterinary education to ensure a high standard of care for this species.

## 1. Introduction

Camelids have been recognized for a long time as essential production animals in many areas of the world. However, seasonal pregnancy and birth rates in traditionally managed herds are generally lower compared to those observed in other domestic species. In camels, the calving rates in extensive herds vary between 25 and 50% [1,2,3]. The estimated seasonal pregnancy rate in Peruvian alpaca and llama herds ranges between 40 and 60% [4,5]. On the other hand, intensively managed herds, under good veterinary care, achieve pregnancy rates of 70% or more both in South American Camelids (SACs) and dromedary camels [6,7]. The discrepancy in reproductive performance between traditionally and intensively managed herds is due to several factors, including nutrition and the prevalence of infectious and parasitic diseases. These factors affect both ovarian activity and the ability to maintain pregnancy (i.e., high incidence of early pregnancy loss) [5,8,9,10,11].

The role of Individual male and female Infertility or subfertility In the overall herd reproductive performance has been understudied until recently. Until the 1990s, most observations on reproductive disorders and infertility in camelids were derived from a single case report or abattoir studies [6,12]. The growing interest in performance camels (racing and show) and SACs outside of South America emphasized the importance of reproductive soundness of the female. Elite females have increased in value and are often maintained in production for a longer time. Additionally, many of the genetically superior females are routinely enrolled in multiple ovulation and embryo transfer programs [8]. These developments have increased the importance of individualized medicine and the need to develop appropriate approaches for the diagnosis of the cause of subfertility and infertility in females.

This paper outlines the approach used by the authors for the diagnostic work-up of infertility or subfertility in female camelids. It provides a retrospective analysis of reproductive disorders in females referred for infertility over the past 35 years. During this time, the average number of cases seen annually is 80 for SACs (USA) and 3000 for dromedary camels (UAE, Qatar, and Morocco). Only cases with complete medical records were included in this report. Incomplete records were primarily due to a lack of precise history (dromedaries) and the owner declining further diagnostics. Dromedary camels will be referred to as ‘camels’ throughout the paper.

## 2. Breeding Soundness Examination of the Subfertile Female

An approach to the investigation of female infertility and subfertility with a complete description of the examination techniques was published in 1997 and updated more recently [7,12]. The female breeding soundness includes a complete medical and reproductive history, followed by a physical examination and a thorough evaluation of the reproductive tract [13]. The main complaints in female camelids with complete medical records are summarized in Table 1. Repeat breeding is the main complaint, followed by early pregnancy loss. These results are consistent with previously published reports in camelids [7,13,14].

A detailed reproductive history is crucial for clinical case definition. Unfortunately, one of the significant constraints in camels is the lack of precise information on breeding management. This differs from SACs in our practice, where the breeding management and results of each mating (i.e., pregnancy diagnosis) are recorded. 

Reproductive examination techniques have improved significantly due to the knowledge gained in reproductive physiology, in particular follicular dynamics, and the use of transrectal ultrasonography, vaginoscopy, as well as endometrial cytology and culture [15,16]. The objective of the initial examination is to rule out major congenital and acquired causes of infertility. This examination is often sufficient to establish a diagnosis in most infertility cases [7,13,14]. It is imperative to inform clients that often the cause of infertility cannot be determined in a single theriogenology visit. Follow-up reproductive evaluations are necessary to ascertain that the female can ovulate and conceive. More advanced techniques such as endometrial biopsy, hysteroscopy, cytogenetics, endocrinology, and laparoscopy are indicated in cases of ovulation failure, fertilization failure, or recurrent early embryo loss [6,7,12].

Complete reproductive examination records were available for 413 SACs and 2387 camels presented for infertility. A diagnosis of the cause of infertility was made for 92.7% of the camel cases and 100% of the SAC cases. The lack of diagnosis in 7.3% of the camel cases can be explained by the inability to perform more advanced examination techniques, such as endometrial biopsy and hysteroscopy. For discussion purposes, reproductive disorders will be divided into two categories: congenital and acquired defects.

## 3. Congenital Defects

All the congenital disorders were observed in infertile maiden females. The main complaints were repeat breeding and visible abnormalities of the external genitalia. Overall, congenital disorders of the reproductive tract were more commonly found in SACs compared to camels. Congenital disorders of the reproductive tract and their relative frequency are presented in Table 2. The most commonly diagnosed disorders were ovarian dysgenesis, segmental aplasia of the tubular genitalia (uterus unicornis, persistent hymen, hydrosalpinx, vaginal aplasia), and atresia vulvi.

**Figure 1:**
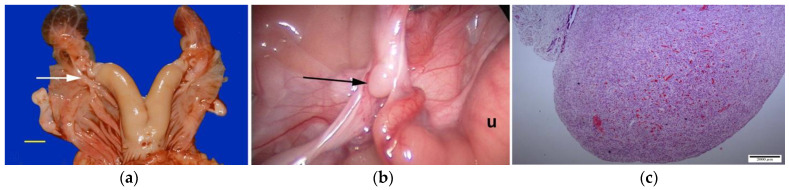
(**a**) Reproductive tract (scale bar: 1 cm) and (**b**) laparoscopic view of an alpaca with ovarian dysgenesis. Arrows indicate ovaries; (**c**) Histology of ovarian hypoplasia in an alpaca (scale bar: 2 mm).

Ovarian dysgenesis/hypoplasia was diagnosed in 36.3% of maiden SACs presented for infertility. This is higher than the 16.8% incidence reported in slaughtered alpacas that were culled for infertility [17]. The high incidence in our data may be due to the inability of referring veterinarians to diagnose these defects. Affected females are generally presented for repeat breeding without evidence of ovulation. In rare cases, the female may be presented due to male rejection in the absence of a pregnancy or a rise in progesterone level. The disorder is characterized by the lack of normal follicular activity, difficulty in visualizing the ovary by ultrasonography, and an infantile reproductive tract [7,18]. Confirmation of the diagnosis is achieved via laparoscopy (Figure 1). Hormonal diagnosis (i.e., serial serum estradiol concentration, anti-mullerian hormone (AMH) concentration) is possible but rarely performed. The ovaries are extremely small, measuring 2 to 3 mm in length. Histologically, this syndrome can be categorized as ovarian hypoplasia (i.e., germ cell deficiency or low germ cell resistance), ovarian dysgenesis (i.e., defective embryonic development of the gonad), or ovarian dysplasia (i.e., abnormal follicular development) [18].

Several chromosomal abnormalities have been associated with ovarian hypoplasia/dysgenesis in SACs, including X0, XX/XY, and XXX [7,19]. A unique cytogenetic abnormality, known as the minute chromosome syndrome, has been observed in alpacas and llamas, affecting over 50% of cases of ovarian dysgenesis. This abnormality is characterized by a significant size discrepancy between chromosome 36 homologs, suspected to result from a translocation [20,21,22].

Ovarian follicular inactivity has been documented in infertile maiden camels; however, the lack of cytogenetic and histological studies cannot confirm its congenital nature as described in SAC [13,14,23].

Cystic formations of the caudal mesonephric tubules (Wolffian ducts), also called paraovarian cysts or paroophoron, are commonly found in all camelids but were not included here because they do not affect fertility. However, they should be differentiated from hydrosalpinx and hydrobursitis, which can impair fertilization and oviductal embryo transport.

Other congenital abnormalities of the ovary include cystic rete ovarii and congenital ovarian teratomas [7,13,24,25,26,27].

**Figure 2:**
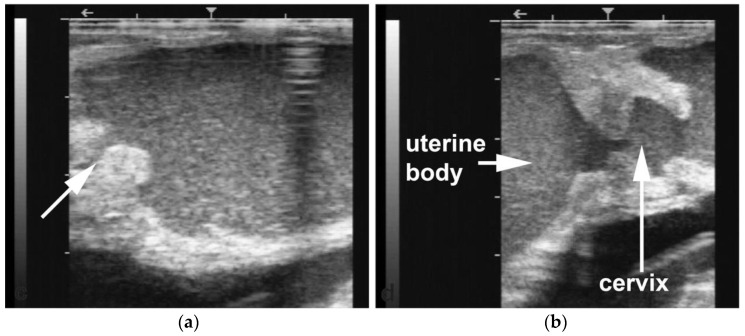
Ultrasonograms of mucometra due to an imperforate hymen in an alpaca. (**a**) cranial vagina (arrow); (**b**) cervical os (arrow) and uterine body.

**Figure 3:**
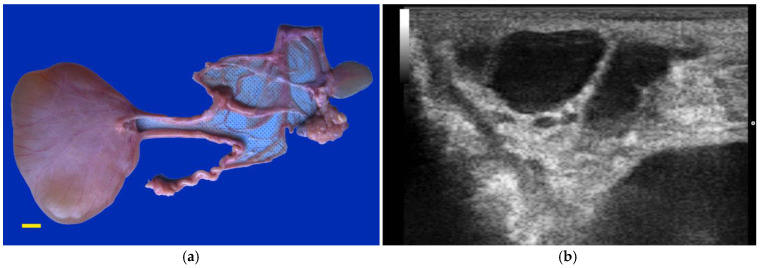
(**a**) Hydrosalpinx due to segmental aplasia in an infertile dromedary female (scale bar: 1 cm); (**b**) Ultrasonographic image of a case of the hydrosalpinx.

Segmental aplasia can affect any part of the tubular genitalia. An imperforate (persistent) hymen is the most common of these abnormalities. Affected females are presented with discomfort or tenesmus during mating. Transrectal ultrasonography reveals mucocolpos and mucometra (Figure 2). Other forms of segmental aplasia of the tubular genitalia include vaginal aplasia, uterus unicornis, and congenital hydrosalpinx due to segmental aplasia of the uterine tube (Figure 3) [6,7,14,28].

**Figure 4:**
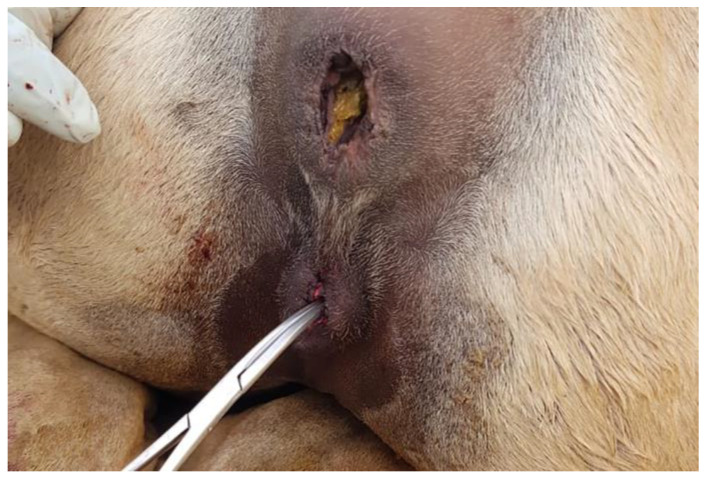
Vulvar atresia in a camel.

The most common abnormality of the caudal genitalia is vulvar atresia (Figure 4). Other abnormalities, including double vagina, double cervix, and vaginal aplasia, have been reported [7,14]. A rare case of uterus didelphis has been described recently in a dromedary [29].

## 4. Acquired Disorders of the Ovaries and Ovarian Bursa

The relative frequency of disorders of the ovaries and uterine tube diagnosed in infertile female SACs and camels is presented in Table 3.

**Figure 5:**
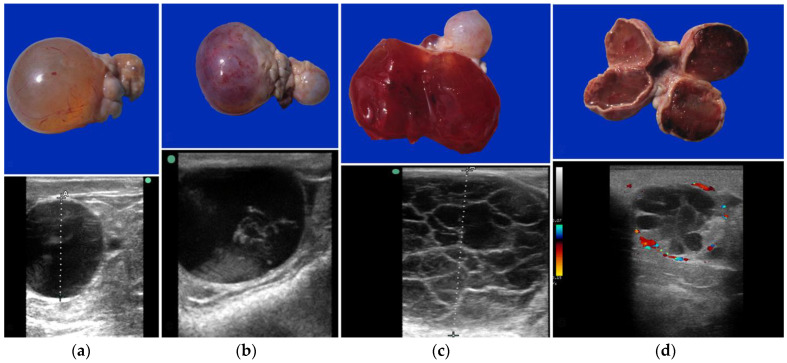
Gross appearance and ultrasonograms of anovulatory follicles in camels. (**a**) Thin-walled anovulatory follicle; (**b**) Thick-walled anovulatory follicle with some fibrin; (**c**) Hemorrhagic anovulatory follicle; (**d**) Luteinized anovulatory follicle.

Cystic structures are the most common ovarian abnormalities found in both SACs and camels. They have been reported previously in SACs [30,31] dromedaries [16,32,33,34], and Bactrian camels [35]. Their role in infertility is not clear. A higher incidence of ovarian cysts was reported in infertile (8.3%) compared to fertile (4.7%) alpacas [17]. A large proportion (30 to 40%) of dromedary females develop anovulatory follicles in the absence of copulation [16]. The incidence of anovulatory follicles varies from 0.9% to 43% depending on the study [26,33,34,36,37,38]. An increase in the incidence of anovulatory follicles was observed in females with clinical endometritis, ovarian hydrobursitis, and vaginal/cervical adhesions [36]. Some females appear to be more susceptible to developing anovulatory follicles. This has been attributed to hormonal imbalances, metabolic disorders, and oxidative stress [33]. However, studies comparing follicular fluid and serum biochemical profiles in females with normal follicles and those with anovulatory follicles did not show any significant differences [39]. On ultrasonography, these structures are large, reaching a diameter of up to 5 cm in llamas and alpacas, and up to 12 cm in dromedaries. They can be completely anechoic or present variable degrees of hemorrhage and luteinization (Figure 5) [16,26]. A rise in serum progesterone level is observed in cases of luteinized hemorrhagic follicles [40]. It is noteworthy that embryo recipients with a luteinized anovulatory follicle can maintain the pregnancy and deliver a live newborn [8].

Lack of ovarian follicular activity was observed in 24.7% of SACs and 29.1% of dromedaries referred for infertility. It is important to note that despite the absence of follicular activity, female camelids can still be receptive to the male. Ovarian inactivity is often associated with loss of body condition during lactation or after a debilitating disease. In camels, seasonal inactivity is marked under harsh conditions and poor nutrition [41]. The incidence of inactive ovaries is substantially higher (57.6% vs. 5.8%) in maiden than in multiparous racing camels [23]. Endocrine abnormalities (i.e., hypothyroidism) and administration of anabolic steroids have been suspected as a potential cause of ovarian inactivity. Aging is also an important factor in the depletion of ovarian follicular reserves. In alpacas, females aged 16 years or older had lower serum AMH concentrations compared to younger females [42].

Oophoritis has been described primarily in slaughtered camels [2,26,34,43] and can be associated with abscesses and hydrobursitis. In cases of oophoritis and ovarian abscess, adhesions may extend to the intestines.

**Figure 6:**
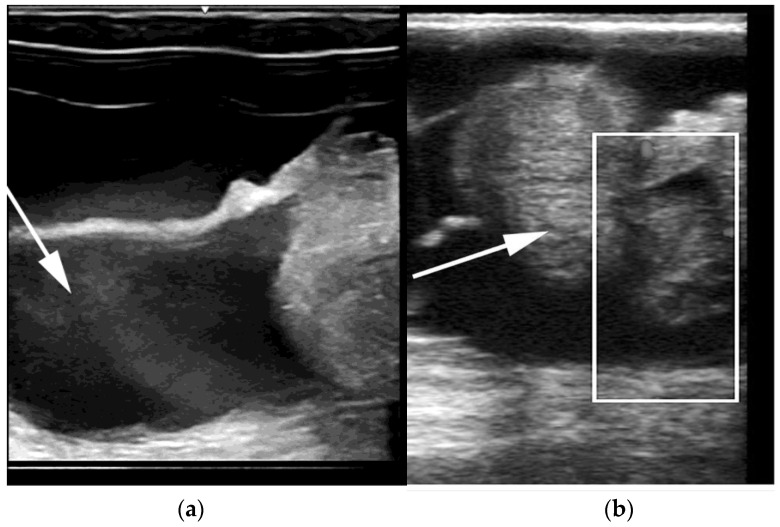
Ultrasonographic images of hydrobursitis in camels. (**a**) Arrow: echogenic fluid within the bursa; (**b**) Arrow: abnormal ovary contained in the fluid-filled bursa.

**Figure 7:**
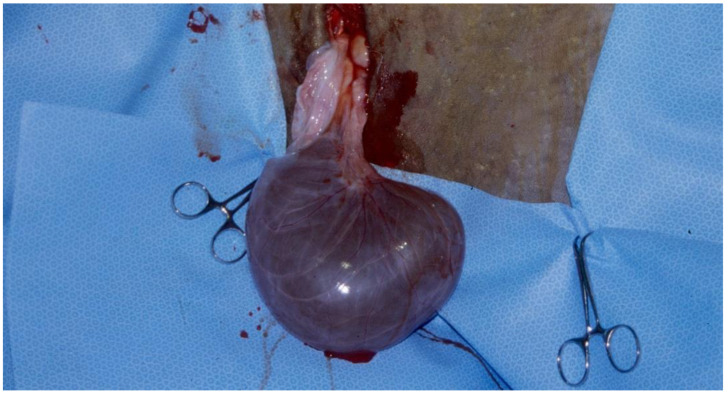
Gross appearance of ovariobursitis surgically exposed by flank laparotomy in a camel.

Hydrobursitis, or ovariobursitis, is a peculiar disorder in camels characterized by the accumulation of varying amounts of fluid within the ovarian bursa [44]. This pathology has been reported worldwide but appears to have a higher incidence in the Middle East [1,23,34,37,44,45]. The etiopathogenesis remains unclear. However, based on biochemical analysis of the fluid content, in some cases, the cause is attributed to recurrent ruptures of hemorrhagic follicles [44,46]. A variety of bacteria have been isolated (*Actinobacillus* spp., *Escherichia coli*, *Klebsiella pneumoniae*, *Pseudomonas aeruginosa*, *Staphylococcus* spp., and *Streptococcus* spp., as well as others) [46,47,48]. More recently, *Chlamydophila abortus* (*Chlamydia abortus*) infection has been demonstrated in several cases of hydrobursitis [49]. The role of *Chlamydia* spp., *Brucella* spp., and *Campylobacter* spp. in this pathology merits further investigation. Clinically, hydrobursitis is suspected when uterine retraction is not possible despite normal size of the uterus and cervix. Confirmation is easily obtained by transrectal or transcutaneous inguinal ultrasonography (Figure 6). Bilateral development is more frequent in females with long-standing infertility (more than 2 years) [44]. The main signs include repeat breeding, early embryo loss, and abortion [1,14,44,45]. Endometritis, uterine adhesions, and pyometra may also be diagnosed in cases of hydrobursitis [14,36,46]. Treatment with oxytetracycline (20 mg/kg IM for 7 days) combined with intrauterine infusion of metacresol sulfonic acid and formaldehyde solution was shown to be effective in restoring fertility if the lesion is small (<3 cm) [50]. Surgical excision of the affected side is the only treatment for large lesions (Figure 7) [44].

Persistence of luteal activity in the absence of pregnancy has been reported following mating-induced ovulation, spontaneous ovulation, and follicular luteinization [51]. An increased incidence is observed in females with endometritis [40].

Ovarian neoplasms are rare in camelids [6,7,52]. Most cases described are from slaughterhouse studies. In camels, the reported incidence of ovarian tumors varies from 0.06% to 6.8% [34,53]. In alpacas, an incidence of 3.2% of all genital abnormalities, with most being teratomas, was reported in Peru [17]. Granulosa cell tumors (GCT) and Granulosa-Theca cell tumors (GTCT) have been described in camels [52,54] and llamas [55,56]. Females may be presented for failure to conceive or for displaying male-like behavior [55,56]. On transrectal palpation, the affected ovary is enlarged with an irregular surface, while the contralateral ovary is inactive. On ultrasonography, the ovary has a multilocular appearance. There are no reports on the use of inhibin or anti-Mullerian hormone (AMH) serum levels for the diagnosis of GTCT in camelids. Metastasis of GTCT is rare, although it was suspected in two llamas [55,56]. Other ovarian tumors reported in camelids include interstitial cells (Leydig cells) [57,58] and Sertoli-Leydig cell tumor (arrhenoblastomas) [59], which are associated with the elevation of serum androgen levels. Other types of ovarian neoplasia have been reported in dromedary slaughterhouse specimens, including papillary cystadenoma, fibroadenoma, luteoma, thecoma, fibroma, cavernous hemangioma, and dysgerminoma [25].

## 5. Acquired Uterine Tube Disorders

**Figure 8:**
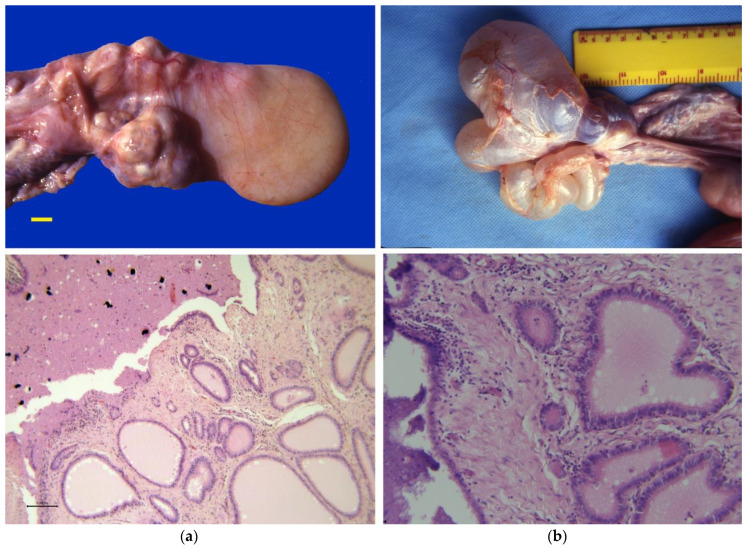
Gross pathology and histopathology of disorders of the uterine tubes in camels. (**a**) Pyosalpinx (scale bar = 1 cm); (**b**) Hydrosalpinx with ovarian-bursal adhesions.

Occlusion of the uterine tubes, resulting in hydrosalpinx and pyosalpinx, has been reported in camelids with chronic salpingitis [6,7,13,37,43,60]. The incidence of uterine tube pathology in slaughterhouse specimens is 0.7% for alpacas [17] and 6.1% for camels [61]. The etiopathogenesis of camelid salpingitis has not been studied, but it is suspected to result from systemic or ascending infection from the uterus following abortion and metritis. In a clinical study, the incidence of pyosalpinx in infertile female camels was 1.8% [54]. Accumulation of fluid or pus in the uterine tube can be diagnosed by transrectal palpation and ultrasonography. This pathology may be associated with ovario-bursal adhesions. Inflammatory changes and the presence of microabscesses are evident on histopathology (Figure 8). Some cases of salpingitis that extend to the uterotubal junction are evident on hysteroscopy. However, many salpingitis cases may go unnoticed in clinical practice [13,62]. Techniques for the evaluation of uterine patency have not been studied in camelids. The authors have used primarily two approaches: embryo collection and surgical normograde flushing of the uterine tube [12].

## 6. Acquired Uterine Disorders

Inflammatory and degenerative uterine pathologies are the most common causes of subfertility or infertility in camelids (Table 4) [6,7].

**Figure 9:**
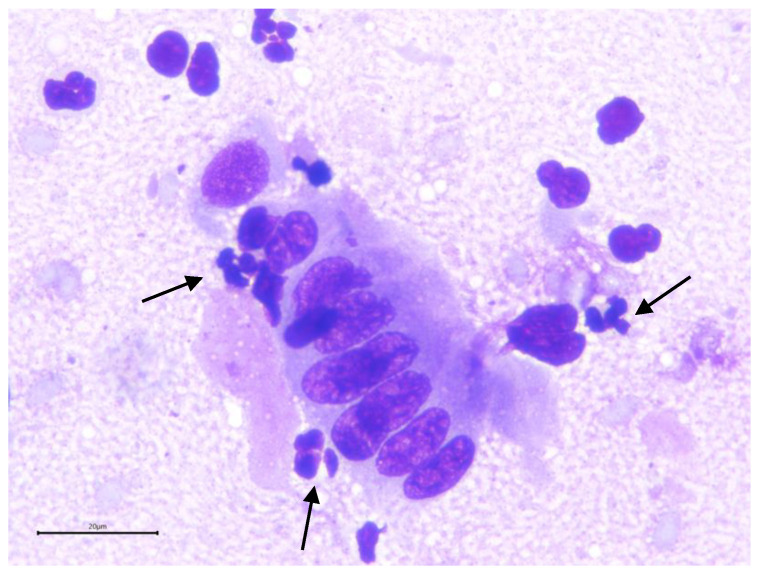
Alpaca endometrial cytology showing polynuclear neutrophils (arrows).

Endometritis has been reported by several authors, mainly in slaughterhouse camel specimens [2,34,63]. Endometrial cytology, culture, and biopsy have all been used for the diagnosis of endometritis in camelids [6,7,12,64,65,66]. The endometrial cytology sample is best obtained with either low-volume flushing or a cytobrush^®^ (Figure 9) [6,12]. Post-mating uterine inflammation is always present in camelids due to intrauterine copulation. Healthy polynuclear neutrophils can be present in the sample for up to 3 days after mating. It is crucial to consider both cytological and bacteriological findings to increase the sensitivity and specificity of diagnosis in cases of subclinical endometritis [6,7,67,68]. Studies on the local defense mechanism of the uterus in camelids are lacking. Our clinical observations suggest that the uterus of camelids has efficient mechanisms to clear this post-breeding inflammation and infection. These mechanisms may fail due to overbreeding, degenerative uterine changes, systemic illness, and inappropriate intrauterine manipulation.

**Figure 10:**
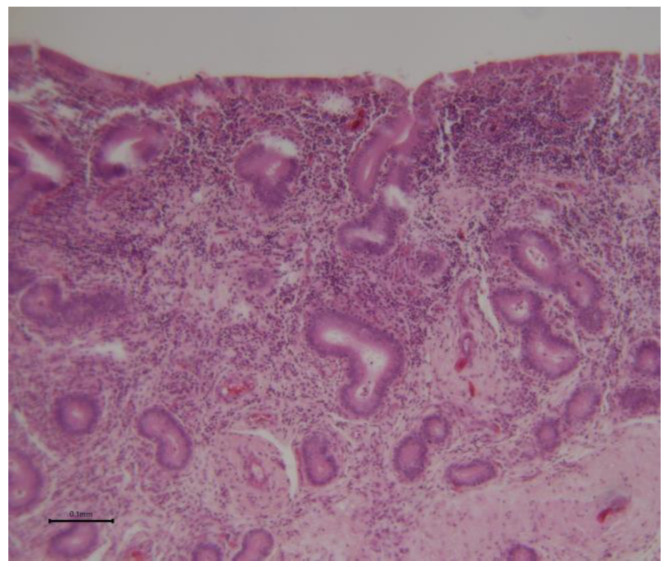
Camel endometrial biopsy exhibiting acute inflammatory reaction (acute endometritis).

The most common bacterial isolates in cases of camelid endometritis are *Streptococcus equi zooepidemicus*, β-hemolytic *Streptococci*, *Enterococcus* spp., coagulase-negative *Staphylococcus* spp., *Proteus* spp., *Enterobacter aerogenes*, *Klebsiella pneumoniae*, *Pseudomonas aeruginosa*, and *Trueperella pyogenes* [14,26,66,67,68,69,70]. Samples from camels should also be examined for *Ureaplasma* spp., *Mycoplasma* spp., and fungi [26,67,69,70,71], and *Campylobacter fetus venerealis*, which has been suspected to cause infertility [66,70,72]. Endometrial biopsy is considered the gold standard for the diagnosis of endometritis and should be an integral part of the evaluation of the barren female (camel [12,13,68,69,73], llama, and alpaca [7,60,74]). The endometrium shows widespread polymorphonuclear neutrophils in acute endometritis (Figure 10).

**Figure 11:**
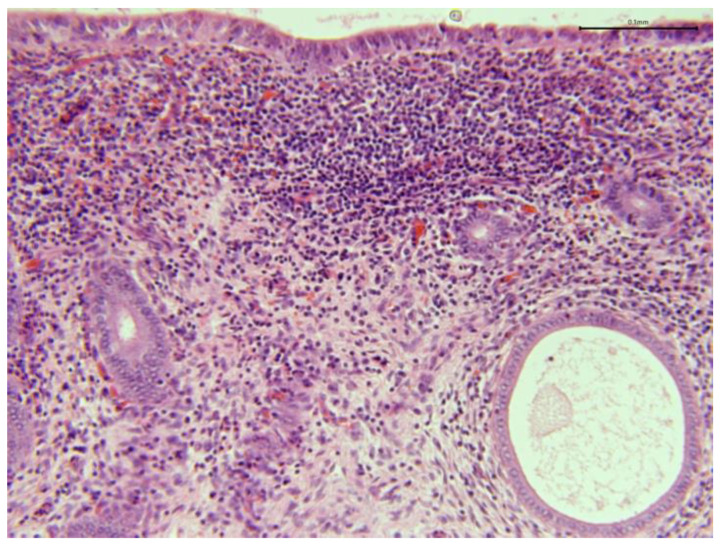
Camel endometrial biopsy exhibiting mixed inflammation in the stratum compactum and periglandular fibrosis with cystic dilation (chronic endometritis).

In chronic endometritis, the infiltration consists predominantly of lymphocytes, and occasional plasmacytes, macrophages, eosinophils, or mast cells (Figure 11).

**Figure 12:**
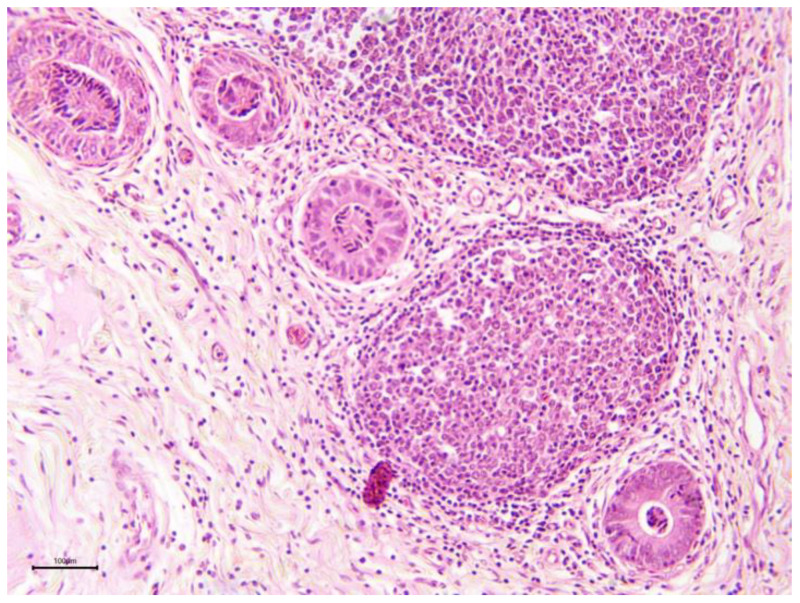
Camel endometrial biopsy exhibiting chronic granulomatous endometritis (scale bar: 100 μm).

A granulomatous chronic endometritis has been described in camels. Similar lesions have been described in cattle with campylobacteriosis, tuberculosis, or chronic fungal infections (Figure 12) [6,12,69,75].

Controlled trials on the treatment of endometritis in camelids are scarce. Therapeutic approaches have been largely adapted from those used in the equine and bovine species. These consist of uterine lavages with saline or weak antiseptic solution, followed by daily systemic or intrauterine antibiotic treatment [6,65]. A first-generation cephalosporin (cephapirin) used for intrauterine infusion in cattle has also been successfully used in camels [76,77]. It is important to note that camelids are food-producing animals in many countries, and veterinarians should adhere to rules of antibiotic stewardship and educate clients about withdrawal times for milk and meat for each drug. Antiseptic solutions formulated for cattle (1% policresulen, 4% metacresol sulphonic acid and formaldehyde, 0.1% solution of acriflavine) have been used successfully to treat uterine infections in camels [78]. Additional therapies used in equine practice, such as uterine infusion with mucolytic or chelating agents (N-acetylcysteine 20%, tris-EDTA, and Tricide^®^) to dissolve biofilm and improve antimicrobial activity, have been used anecdotally for the treatment of endometritis in camelids. A preliminary clinical trial was conducted on eight camels using ozone foam for the treatment of chronic endometritis with satisfactory results (A. Tibary, personal observation). Future studies should include the use of antimicrobial peptides and platelet-rich plasma for the treatment of endometritis in camelids.

The incidence of endometritis can be reduced by minimizing the risk of contamination of the uterus during mating, obstetrical interventions, and other reproductive manipulations. In our experience, the leading causes of uterine infections are improper intrauterine treatments and overbreeding. The reproductive tract microbiome and the role of the vaginal flora changes in the pathophysiology of endometritis in camelids merit more investigation [79].

Other uterine disorders include degenerative changes, pyometra/mucometra, and uterine tumors. Chronic degenerative changes or endometrosis are a common histopathological finding in camelids with a history of repeat breeding or recurrent pregnancy losses. Its pathophysiology remains poorly studied in camelids, but it is often a consequence of chronic uterine inflammation. Although a classification of endometrial biopsy has been published for camelids, there is no data correlating this biopsy grade to the probability of a female carrying a pregnancy to term [7,12,74].

**Figure 13:**
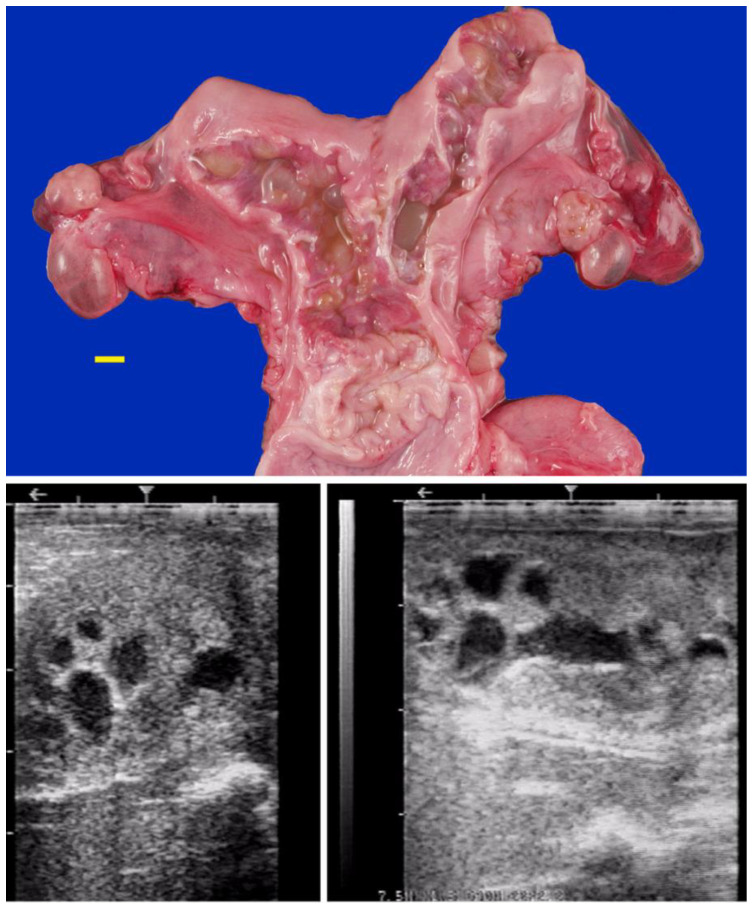
Gross pathology (scale bar: 1 cm) and ultrasonogram of intraluminal uterine cysts due to intraluminal uterine adhesions in an alpaca.

Pyometra and non-congenital mucometra are often due to cervical or vaginal adhesions resulting from poor handling of dystocia or fetotomy [7,14,63]. Uterine cysts and intraluminal adhesions have also been observed following dystocia or postpartum uterine prolapse (Figure 13).

Uterine neoplasms are rare in camelids. Reported cases include adenocarcinomas, leiomyomas, and hemangiomas [6,7,24,52,74,80]. Females are presented for repeat breeding or post-mating vaginal bleeding. Most cases are suspected on transrectal palpation or ultrasonography and confirmed by biopsy or postmortem examination of the uterus. Long-term prognosis for all uterine neoplasms is poor. Metastases to the lymph nodes, mesentery, and lungs have been reported in SACs [24,80].

Other uterine diseases found in camelids include uterine cysts, uterine abscesses, peri-uterine adhesions, intraluminal adhesions, uterine wall perforation, foreign bodies, polyps, fibromas, and endometrial gland dysgenesis [6,7,28,53]. Most of these disorders are suspected on transrectal palpation and ultrasonography, and can be confirmed by uterine biopsy, hysteroscopy, or laparoscopy. They generally carry a poor prognosis for fertility [6].

## 7. Acquired Disorders of the Cervix

The most common acquired pathological condition of the cervix diagnosed in our practice is cervical trauma (adhesions, tears) (Table 5). These lesions are often the result of unattended dystocia and inadequate obstetrical or gynecological manipulations [7,14,64]. Females with these cervical pathologies are presented for repeat breeding or recurrent pregnancy loss. Mucometra is always present in cases of non-patent cervixes [13,53,64,69,81]. Cervical fibrosis and stenosis occur in some cases and are suspected when catheterization of the cervix is not possible. In alpacas, hysteroscopy or contrast fluoroscopy are the best methods for diagnosis [7]. Breaking cervical adhesions manually has been suggested as a treatment [81]. However, the adhesions tend to recur and are often more severe. Placing a cervical stent may provide a better way to prevent large fluid buildup and help maintain the reproductive capacity of valuable females, enabling them to be used for oocyte collection and in vitro embryo production.

Cervicitis is often associated with uterine infections [63,69]. It should be differentiated from the normal hyperemia and slight irritation found immediately following breeding.

Cervical neoplasms are sporadic, but a few cases, mainly adenocarcinomas and leiomyomas, have been reported [52,80].

## 8. Acquired Disorders of the Vulva and Vagina

In camelids, acquired disorders of the vulva and vagina include inflammation (vulvitis, vaginitis), trauma, and neoplasia (Table 6). Vulvar edema and vulvitis may result from irritation (mating, diarrhea, or chemical) or parasite infestation (i.e., mange, ticks, myiasis due to *Wohlfahrtia magnifica*) [6,12,82]. A form of viral granular vulvovaginitis has been described in camels and attributed to Bovine herpes virus-1 [83]. Vulvovaginitis is also observed in camel pox virus infections. Non-specific or zinc-responsive vulvar and perineal hyperkeratosis is often seen in alpacas and llamas.

Vaginitis is uncommon and associated with poor breeding management and inadequate gynecological manipulations. Necrotic vaginitis is often seen following dystocia and may progress to vaginal adhesions leading to mucometra [7,12,81]. A few cases of vaginal tumors (leiomyosarcoma in an alpaca [84], adenocarcinoma in camels [85]) have been reported.

**Figure 14:**
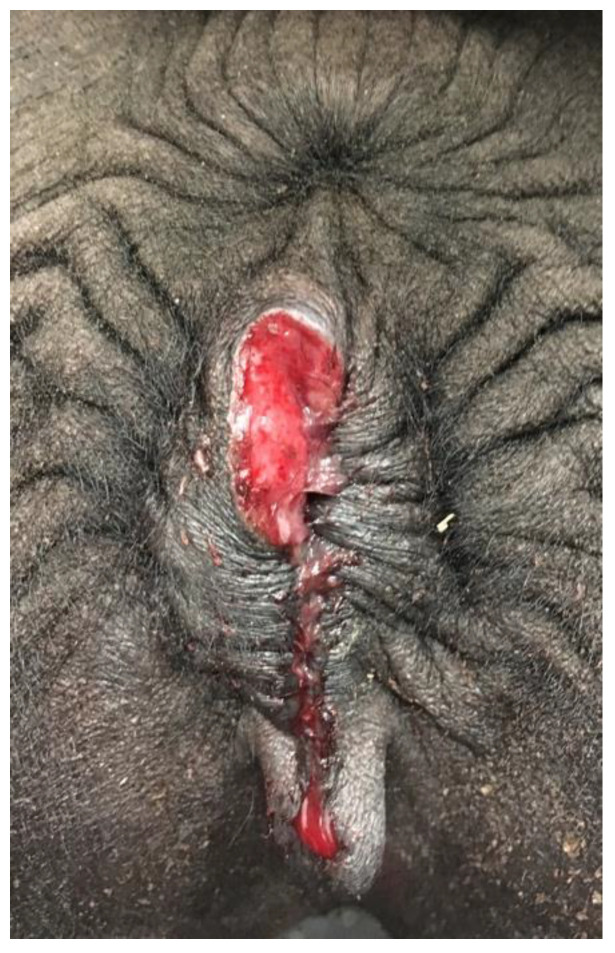
Vulvar squamous cell carcinoma in a llama.

Rectovaginal tears are common following dystocia due to the small perineal body in camelids. In camels, lesions on or around the vulva may be due to “traditional infertility treatment” (firing, clitoridectomy) [12]. Vulvar neoplasia is rare. Two cases of vulvar squamous cell carcinoma have been diagnosed by the authors in llamas (Figure 14).

## 9. Conclusions

This article aimed to provide an overview of the advances in the diagnosis of reproductive disorders in female camelids. The distribution of disorders shown here may not accurately represent their actual occurrence in the general population, as our cases are drawn from referrals to specialized centers and are thus selected samples. However, our data illustrate the wide range of disorders that may affect the camelid reproductive system. Significant progress has been made in developing methods to diagnose these disorders. However, there is a considerable discrepancy between the standard of care for SACs and camels. In camels, advanced techniques such as endometrial biopsy and endocrinology are often not used, and cytogenetic evaluations are entirely lacking.

In our experience, camelid veterinary care is often not included in the standard veterinary curriculum in many countries. This deficit frequently results in a significant shortage of veterinarians equipped with the clinical and laboratory skills necessary to perform accurate reproductive evaluations. Failure to properly diagnose reproductive disorders can have serious consequences for animal welfare, especially for females with congenital defects.

Uterine disorders, endometritis in particular, appear to be a major contributor to reproductive loss. Research on camelid uterine defense mechanisms, the role of the reproductive microbiome, and studies on factors predisposing to uterine infection in these species are urgently needed. In many regions of the world, improper diagnosis of uterine infection leads to the overuse of antibiotics with subsequent increases in the risk for antibiotic resistance, which poses a significant health concern for both animal and human populations.

Veterinarians should also consider assisted reproductive technologies (i.e., embryo collection, oocyte collection, and in vitro fertilization) as alternative methods for reproduction of valuable females with severe acquired uterine and utero-tubal disorders. 

## Figures and Tables

**Table 1:** Proportion of different reproductive complaints in female camels (*n* = 542) and South American camelids (SACs) (*n* = 2495).

Primary Complaint	SACs ** (%)	Camels * (%)
Repeat breeding	2003 (80.30)	411 (75.83)
Early pregnancy loss	448 (18.00)	88 (16.23)
Behavioral problems	16 (0.64)	15 (2.77)
Visible genital abnormalities	28 (1.12)	28 (5.17)

* Cases seen in UAE, Qatar, Morocco, ** Cases seen in USA.

**Table 2:** Congenital abnormalities of the reproductive tract diagnosed in infertile SACs (*n* = 102) and camels (*n* = 25).

Congenital Abnormality	SACs (%)	Camels (%)
Atresia vulvi/vulvar hypoplasia	23 (22.54)	3 (12.00)
Double cervix	2 (1.96)	2 (8.00)
Double vagina	2 (1.96)	1 (4.00)
Hydrosalpinx (segmental aplasia)	2 (1.96)	5 (20.00)
Imperforate hymen	15 (14.70)	3 (12.00)
Intersex/ambiguous gender	5 (4.90)	1 (4.00)
Ovarian dysgenesis	37 (36.27)	6 (24.00)
Ovarian teratoma	1 (0.98)	1 (4.00)
Vaginal aplasia	10 (9.80)	-
Uterus unicornis	5 (4.90)	3 (12.00)
Total number of animals	102	25

**Table 3:** Distribution of acquired disorders of the ovaries, ovarian bursa, and uterine tube in SACs and camels presented for infertility.

Abnormality	SACs (%)	Camels (%)
Anovulatory/Hemorrhagic follicles	42 (47.19)	669 (54.34)
Hydrobursitis	3 (3.37)	149 (12.10)
Ovarian adhesions	3 (3.37)	8 (6.49)
Ovulation failure	8 (8.98)	10 (0.81)
Ovarian inactivity	22 (24.71)	358 (29.08)
Ovarian tumors	2 (2.24)	4 (0.32)
Persistent luteal structures	16 (17.97)	33 (2.68)
Pyosalpinx/salpingitis	1 (1.12)	82 (6.66)
Total number of animals	89 *	1231 *

* The total percentage is greater than 100% because some animals presented a combination of abnormalities.

**Table 4:** Distribution of acquired uterine disorders in SACs and camels.

Abnormality	SACs (%)	Camels (%)
Endometrial degeneration	33 (20.00)	371 (40.77)
Endometritis	84 (50.91)	501 (55.05)
Intestinal-uterine adhesions	2 (1.21)	14 (1.54)
Luminal adhesions	12 (7.27)	3 (0.33)
Mucometra-pyometra	18 (10.91)	10 (1.10)
Mummification	1 (0.61)	1 (0.11)
Neoplasia	5 (3.03)	1 (0.11)
Uterine abscess	2 (1.21)	6 (0.66)
Uterine cysts	8 (4.85)	4 (0.44)
Total number of animals	165	910

**Table 5:** Distribution of acquired disorders of the cervix in SACs and dromedary camels.

Abnormality	SACs (%)	Camels (%)
Cervical stenosis/adhesions	10 (34.48)	6 (54.54)
Cervicitis	7 (24.14)	3 (27.27)
Cervical tears	12 (41.38)	2 (18.18)
Total number of animals	29	11

**Table 6:** Distribution of acquired disorders of the vaginal and vulva in SACs and dromedary camels.

Abnormality	SACs (%)	Camels (%)
Recto-vaginal tears	11 (39.28)	10 (27.77)
Squamous cell carcinoma	2 (7.14)	-
Vaginal adhesions	13 (46.42)	6 (16.66)
Vaginitis	1 (3.57)	2 (5.55)
Vulvitis	1 (3.57)	18 (50.00)
Total number of animals	28	36

## Data Availability

The original contributions presented in this study are included in the article. Further inquiries can be directed to the corresponding author.

## Abbreviations

SACs	South American Camelids
USA	United States of America
UAE	United Arab Emirates
AMH	Anti-Mullerian Hormone
SPP	Species
GCT	Granulosa Cell Tumor
GTCT	Granulosa Theca Cell Tumor
EDTA	Ethylenediaminetetraacetic acid
IE	Id Est
